# A Case of Dapsone-Induced Severe Agranulocytosis Causing Life-Threatening Skin Sepsis in a Sri Lankan Child with Borderline Leprosy: A Success Story!

**DOI:** 10.1155/2019/2314379

**Published:** 2019-05-06

**Authors:** Meranthi Fernando, Imalke Kankananarachchi, Pratheep Navabalasooriyar, Bhagya Herath, Pushpa Punchihewa

**Affiliations:** ^1^University of Kelaniya, Colombo, Sri Lanka; ^2^University of Ruhuna, Matara, Sri Lanka; ^3^Lady Ridgeway Hospital for Children, Colombo, Sri Lanka

## Abstract

Leprosy is a common skin disease in Sri Lanka which is being increasingly diagnosed due to the existing successful public health programme. Dapsone is a drug which holds unique pharmacological properties where it serves as both anti-inflammatory and antimicrobial agents. Of its main adverse effects, agranulocytosis is a serious consequence which is reported mainly in adults and elderly. We report a 7-year-old child who sustained life-threatening skin and subcutaneous tissue sepsis because of dapsone-induced agranulocytosis. Besides, this case highlights the importance of meticulous monitoring of cell counts due to the risk of neutropenia and the natural history of cell recovery following occurrence of neutropenia. Though high mortality rate has been described in most of the similar cases reported, the child we describe made a complete recovery following severe neutropenic sepsis.

## 1. Introduction

Leprosy is a common dermatological disorder among adults and in children in Sri Lanka. It is increasingly being detected due to raised awareness [1]. Dapsone (4,4′-diaminodiphenylsulfone) has been used as an antileprosy agent since the 1940s [2]. In addition, it is being used in multiple other dermatological conditions [3]. Though dapsone is a very effective drug, it carries a unique adverse effect profile [4]. Agranulocytosis is a rare, serious adverse effect following dapsone therapy which carries a high mortality rate [4]. There are no cases reported on dapsone-induced agranulocytosis in Sri Lankan paediatric population. Moreover, a limited number of cases have been reported in children internationally. Here, we report a 7-year-old girl who sustained life-threatening skin sepsis secondary to dapsone-induced agranulocytosis and recovered eventually [5].

## 2. Case Report

A 7-year-old girl presented with fever and swelling of the face and neck for 2 days. Symptoms were gradually progressive with dysphagia and difficulty in breathing.

**Figure 1:**
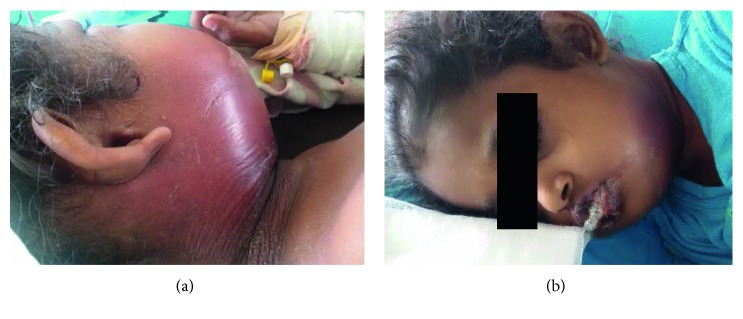
Skin sepsis with severe mucositis.

Examination revealed an ill, febrile child with swelling of the face and neck with associated cellulitis. Severe mucositis was noted with trismus and drooling of saliva (Figure 1). She had dental caries. Clinical condition deteriorated with severe cellulitis and formation of a deep-seated abscess in the submandibular region and resulted in stridor.

**Figure 2:**
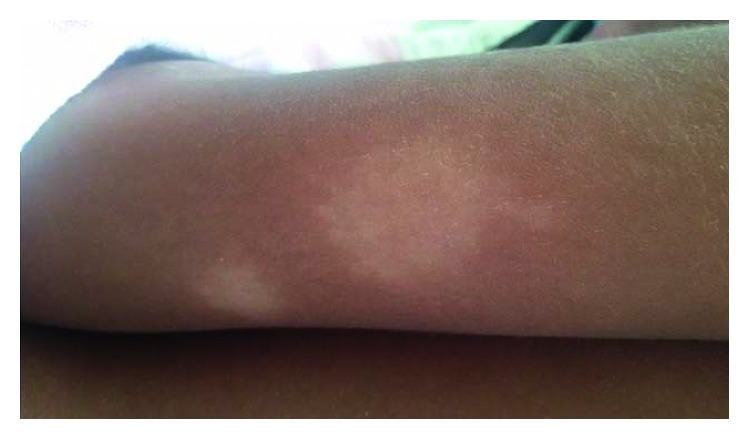
Two hypopigmented skin lesions over the left upper arm.

There were two hypopigmented skin lesions over the left arm with loss of thermal sensations which raised the suspicion of leprosy (Figure 2). No thickened palpable nerves were identified. The underlying diagnosis of leprosy was apparent with direct questioning, and it was revealed that the child had been on rifampicin and dapsone for 2 months. Unfortunately, no cell counts were monitored since commencement of antileprosy medications.

Investigations revealed a white blood cell (WBC) count of 1,000/mm^3^ with an absolute neutrophil count (ANC) of zero. Blood picture revealed dapsone-induced changes with numerous bite cells, blister cells, and agranulocytosis. Bone marrow examination was a bloody tap, and it was not repeated as the child improved with supportive care.

Inflammatory markers showed a CRP level of 220 with an ESR of 70 at the 1^st^ hour. Blood culture was sterile. Liver and renal functions were normal. Serial USS showed deep-seated abscesses with overlying skin oedema in the submandibular region bilaterally.

Management included immediate cessation of dapsone with commencement of broad-spectrum antibiotics. Repeated incision and drainage were required to drain the abscesses. Nebulised adrenaline and IV dexamethasone were used to manage stridor and airway compression. Granulocyte colony-stimulating factor (GCSF) was used initially to manage neutropenia to which she had a poor response. Thus, buffy coat was transfused as per management of any other case of neutropenia [6].

Her ANC rose up to 1500, following 5 days of admission, and she made a complete recovery (Table 1).

## 3. Discussion

Dapsone has been widely used to treat many dermatological and autoimmune conditions due to its antibacterial and anti-inflammatory actions [7]. Inhibition of bacterial folate synthesis is the mechanism of its antibacterial property; however, there is no clear explanation for its anti-inflammatory action [6].

The prevalence of dapsone-induced agranulocytosis is 0.2–0.4% [8]. And it is possibly due to its idiosyncratic action. Other common haematological side effects such as haemolytic anaemia and methemoglobinemia are dose dependent [7].

Agranulocytosis due to dapsone therapy was described among 16 US soldiers in Vietnam when they were treated for prophylaxis of malaria. Majority of them developed agranulocytosis within 1 to 3 months of the therapy [9]. Similarly, in this case, the onset of neutropenia was after 2 months of treatment. The common clinical manifestations were fever, lymphadenitis, tonsillitis, and septicaemia where the mortality rate was nearly 50%. Though this child made a quick recovery with complete normalisation of ANC, there had been cases where prolonged neutropenia was observed even after withdrawal of the drug. It could possibly be due to the extensive protein-binding property of the drug and might be related to enterohepatic circulation [4].

Management of dapsone-induced agranulocytosis includes prompt cessation of therapy and commencement of broad-spectrum antibiotics as per management of febrile neutropenia [4]. GCSF is indicated when ANC is less than 0.1 × 10^9^/L.

Agranulocytosis should actively be sought in patients on dapsone irrespective of the underlying diagnosis. Full blood count should be performed fortnightly during the first 3–6 months followed by once in 2-3 months subsequently [10]. Furthermore, repeated health education messages are prudent to make the primary health care workers and patients vigilant in detecting this important adverse effect as early presentation will be life-saving.

## Figures and Tables

**Table 1:** Serial blood counts.

Day	1	2	3	5	6
WBC	1000	400	600	7.3	24.7
N (%)	0	3.5	4.6	22	68
L (%)	80	87	71	75	17
ANC	0	14	27	1606	16796
Hb	9.5	8.6	7.2	13.3	14.2
PLT	289	296	192	235	289
